# MicroRNAs: new biomarkers and therapeutic targets after cardiac arrest?

**DOI:** 10.1186/s13054-015-0767-2

**Published:** 2015-02-11

**Authors:** Yvan Devaux, Pascal Stammet, Hans Friberg, Christian Hassager, Michael A Kuiper, Matt P Wise, Niklas Nielsen, for the Biomarker subcommittee of the TTM trial (Target Temperature Management After Cardiac Arrest, NCT01020916)

**Affiliations:** Laboratory of Cardiovascular Research, Luxembourg Institute of Health, L-1526 Luxembourg, Luxembourg; Department of Anaesthesia and Intensive Care Medicine, Centre Hospitalier, L-1445 Luxembourg, Luxembourg; Skane University Hospital, Lund University, SE-205 02 Malmo, Sweden; Department of Cardiology, The Heart Center, Rigshospitalet, DK - 2100 Copenhagen, Denmark; Department of Intensive Care Medicine, Medical Center Leeuwarden, 8934 AD Leeuwarden, The Netherlands; Adult Critical Care, University Hospital of Wales, Cardiff, CF14 4XW UK; Helsingborg Hospital, Lund University, S-251 87 Helsingborg, Sweden

## Abstract

Despite advances in resuscitation medicine, including target temperature management as part of post-cardiac arrest care, many patients will have a poor neurological outcome, most often resulting in death. It is a commonly held belief that the ability to prognosticate outcome at an early stage after cardiac arrest would allow subsequent health care delivery to be tailored to individual patients. However, currently available predictive methods and biomarkers lack sufficient accuracy and therefore cannot be generally recommended in clinical practice. MicroRNAs have recently emerged as potential biomarkers of cardiovascular diseases. While the biomarker value of microRNAs for myocardial infarction or heart failure has been extensively studied, less attention has been devoted to their prognostic value after cardiac arrest. This review highlights the recent discoveries suggesting that microRNAs may be useful both to predict outcome and to treat patients after cardiac arrest.

## Introduction

Although half of the patients resuscitated from cardiac arrest survive without major neurological sequelae, the other half die and some other survivors have severe neurological impairment. This is despite the widespread use of therapeutic hypothermia and its potential neuroprotective effects. Early outcome prognostication of patients resuscitated from cardiac arrest is challenging, mostly due to the paucity of accurate tools. The implementation of mild induced hypothermia has complicated matters further because the metabolism of sedatives is unpredictable and the use of muscle paralysis may confound prognostication [1]. Until recently, clinical neurological examination and neurophysiological tests performed several days after the arrest were the best indicators of outcome [2,3]. The use of circulating biomarkers such as neuron-specific enolase (NSE) improves outcome prediction on a group basis and has been recommended in clinical practice [4,5]. However, the discriminative ability of these tests is sub-optimal in individual patients and management of individuals could potentially benefit from new biomarkers.

Discovered in 2001 in *Caenorhabditis elegans* [6-8], microRNAs (miRNAs) have attracted great interest in the scientific community. The discovery of their presence and stability in the bloodstream [9,10] revealed their potential as novel disease biomarkers. Multiple groups have addressed the utility of circulating miRNAs as biomarkers of cardiovascular diseases. The vast majority of studies focused on myocardial infarction and heart failure. However, the potential of miRNAs to be used as biomarkers of cardiac arrest has received less attention and, until recently, was totally neglected.

In this article, we first review the current knowledge on available biomarkers used to predict outcome after cardiac arrest. Then, we discuss why it is critical to identify new biomarkers, and how these new tools may enable improvements in health care and outcome of patients after cardiac arrest. Finally, we present recent data suggesting that miRNAs might be useful biomarkers and therapeutic targets in this setting.

## Current biomarkers: limitations

In order to predict outcome of patients with post-anoxic coma after circulatory arrest, biomarkers of neuronal damage have been extensively studied. Creatine phosphokinase brain-brain (CK-BB), NSE and the astroglial protein S100 calcium binding protein B (S100B) [11-14] have been evaluated in cerebrospinal fluid and blood of cardiac arrest patients. We have recently shown that combining serum levels of S100B and bispectral index monitoring accurately predicts outcome after cardiac arrest [15]. For a systematic review of the existing literature on biomarkers of cardiac arrest, see [16,17].

The cutoff values of CK-BB and S100B required to obtain sufficient specificity (and therefore sufficiently low false positive rates) are substantially elevated. Consequently, these biomarkers have low sensitivity, and are of limited prognostic value.

Although NSE was identified in the late 1980s as a potential marker of neurological outcome after cardiac arrest [18], its clinical utility is still a subject of debate. NSE is an isoenzyme of the glycolytic enzyme enolase (2-phospho-D-glycerate hydrolase) and is mostly of neuronal and neuroendocrine origin. Its levels are elevated after ischemic stroke, intracerebral hemorrhage, and traumatic and ischemic brain injury, rising just hours after neuronal damage. These properties make NSE a potential useful biomarker for neurologic outcome after cerebral injury. The 2006 American Academy of Neurology guidelines on prediction of outcome in comatose survivors after cardiopulmonary resuscitation advocated the use of NSE as a biomarker to estimate neurologic outcome with a cutoff value of >33 μg/L, which provided a false positive rate of 0% (95% confidence interval 0 to 3%) [3,19]. However, more recent studies do not support this guideline. Grubb and colleagues [20] found a NSE cutoff value of >71.0 μg/L 24 to 48 hours after cardiopulmonary resuscitation resulted in a false positive rate of 0% (95% confidence interval 0 to 43%) and a sensitivity of 14%. Other studies showed cutoff values of 30 to 80 μg/L for poor neurologic outcome and death [21,22]. Krumnikl and colleagues [23] published a case report of a patient with good neurologic outcome after a period of extended in-hospital cardiopulmonary resuscitation with a highest NSE value of 116.8 μg/L. Interestingly, hypothermia may affect serum levels of NSE. Tiainen and colleagues [24] found that the cutoff value of NSE, 48 hours after cardiopulmonary resuscitation and target temperature management, needed to be two to three times higher compared with patients not undergoing mild induced hypothermia (>25 versus 8.8 μg/L). Steffen and colleagues [25] also found higher cutoff values after hypothermia (NSE 78.9 versus 26.9 μg/L). In contrast, Wolff and colleagues [26] reported lower cutoff values after hypothermia. In addition, there is presently a lack of standardization of the measurements of NSE. Several available laboratory tests show variability of up to 40% between NSE values on the same samples [27].

Interpreting data from biomarker studies is confounded by differences in study design, inclusion/exclusion criteria of patients, duration of treatment, time of sampling and laboratory evaluation, as well as differences in evaluated endpoints of treatment, making it difficult to compare studies in a systematic review or a meta-analysis in a meaningful way. Other more recently described biomarkers such as procalcitonin [28-30], glial fibrillary acidic protein [31], heparin binding protein [32] and brain-derived natriuretic factor [33] face the same methodological issues and further large scale studies with an accurate methodology are warranted.

Therefore, there is an urgent need for identifying novel biomarkers that can guide patient management at an early stage after cardiac arrest.

## How will new biomarkers allow for patient-oriented treatment and improvement of outcome?

Patients who remain unconscious following an out-of-hospital cardiac arrest despite the return of spontaneous circulation utilize considerable health care resources during the first days of admission to the hospital. Patients often require immediate coronary intervention to establish revascularization followed by mechanical ventilation in intensive care where they are treated with mild induced hypothermia, frequently requiring sedation and muscle paralysis. Cardiogenic shock and multiple organ failure may further complicate the clinical course of some survivors, necessitating high dose inotropic drugs, vasopressors, renal replacement therapy, intra-aortic balloon pumps, mechanical assist devices or extra-corporeal membrane oxygenation [34,35]. Despite early aggressive therapy, many of these patients will not survive due to irreversible cerebral damage caused by the initial insult.

New biomarkers may be very valuable if they have sufficient prognostic power when measured early after cardiac arrest. Health care resources may then be applied to patients who are most likely to benefit and futile care for those patients with irreversible severe cerebral damage can be avoided. Furthermore, patients’ relatives, who often experience long delays before receiving reliable information about prognosis, may be informed and guided early.

## Emerging biomarkers: microRNAs

miRNAs are short (around 21 nucleotides) non-protein-coding RNA molecules that are evolutionarily conserved and ubiquitously expressed, albeit with a degree of tissue specificity. Since the first version of *The miRBase Sequence Database* [36] in December 2002, the number of known miRNAs has continued to grow. To date, as many as 30,424 mature miRNAs have been characterized in 206 species, with 2,578 in humans (release 20 June 2013).

**Figure 1:**
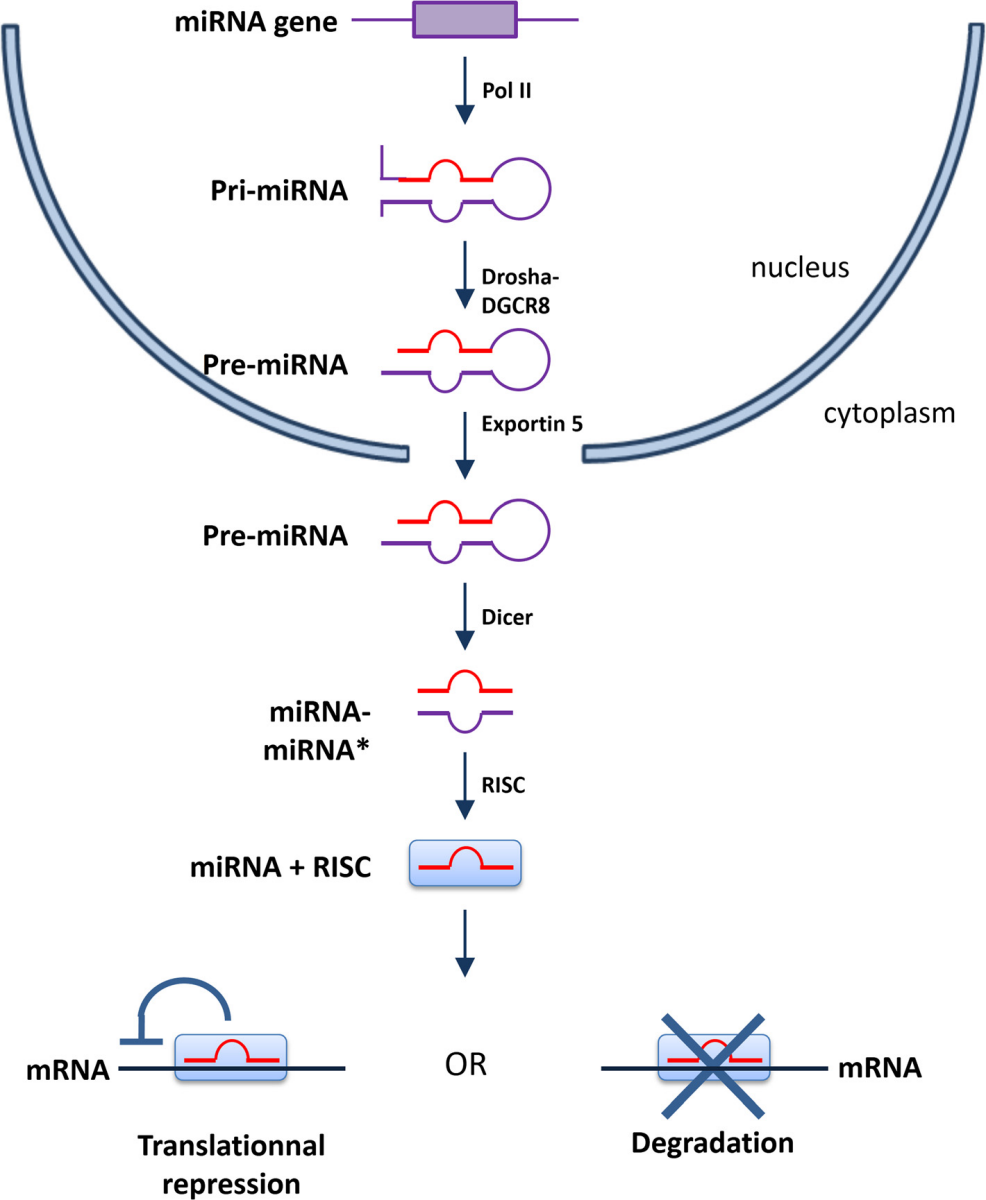
**MicroRNA biogenesis.** miRNA, microRNA; Pol II, polymerase II; RISC, RNA-induced silencing complex. Adapted from Goretti and colleagues [81].

Although miRNAs do not encode proteins, their role in gene regulation, and thereby in protein expression, is significant (Figure 1). Synthesized in the nucleus as primary miRNAs by the RNA polymerase III enzyme complex, they are cleaved by a second enzyme complex called Drosha to generate precursor miRNAs, which are exported to the cytoplasm to be finally cleaved by Dicer to form mature miRNAs. Mature miRNAs bind to target mRNA species and prevent their translation into proteins, either by induction of mRNA degradation by RNA-induced silencing complex when there is a perfect match between miRNA sequence and target mRNA, or by translational blockade when the two sequences are mis-matched. In mammals, miRNAs predominantly regulate gene expression by induction of mRNA degradation [37]. This property allows miRNAs to regulate developmental, physiological, as well as pathophysiological processes [38]. In the heart, miRNAs have been shown to regulate many functions, such as apoptosis, angiogenesis, contractility, and hypertrophy [39]. Similarly, miRNAs are abundantly expressed in the brain [40], where they play key roles in development, plasticity and disease evolution [41].

**Figure 2:**
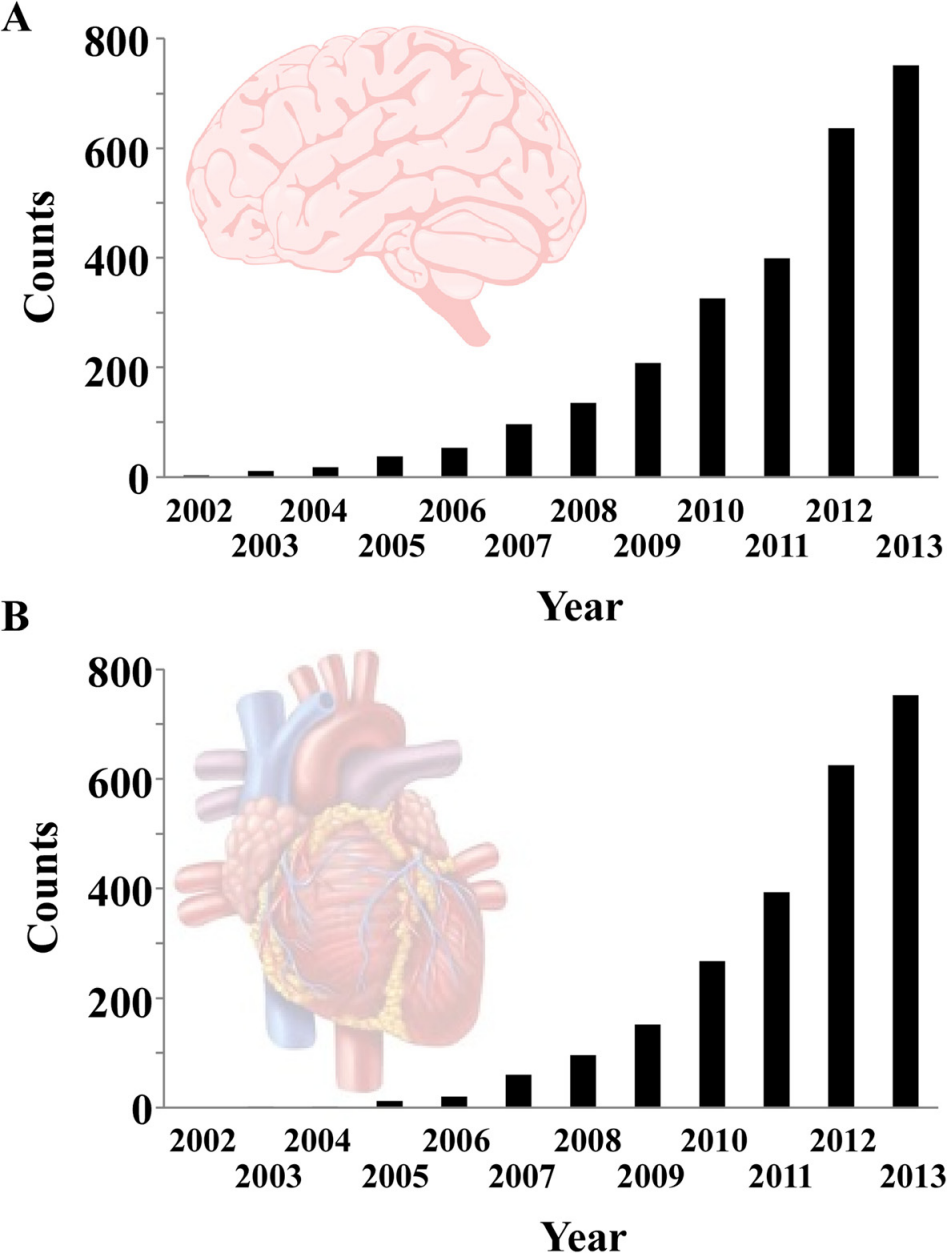
**PubMed literature searches of research articles related to microRNAs. (A)** Evolution of the number of articles related to microRNAs (miRNAs) in the neurological system. Literature search was performed in PubMed using the query: (microrna OR mirna OR “micro-rna”) AND (brain OR neuron OR neurological OR cerebral). **(B)** Evolution of the number of articles related to miRNAs in the cardiovascular system. Literature search was performed in PubMed using the query: (microrna OR mirna OR “micro-rna”) AND (heart OR cardiac OR myocardial OR cardiovascular).

**Figure 3:**
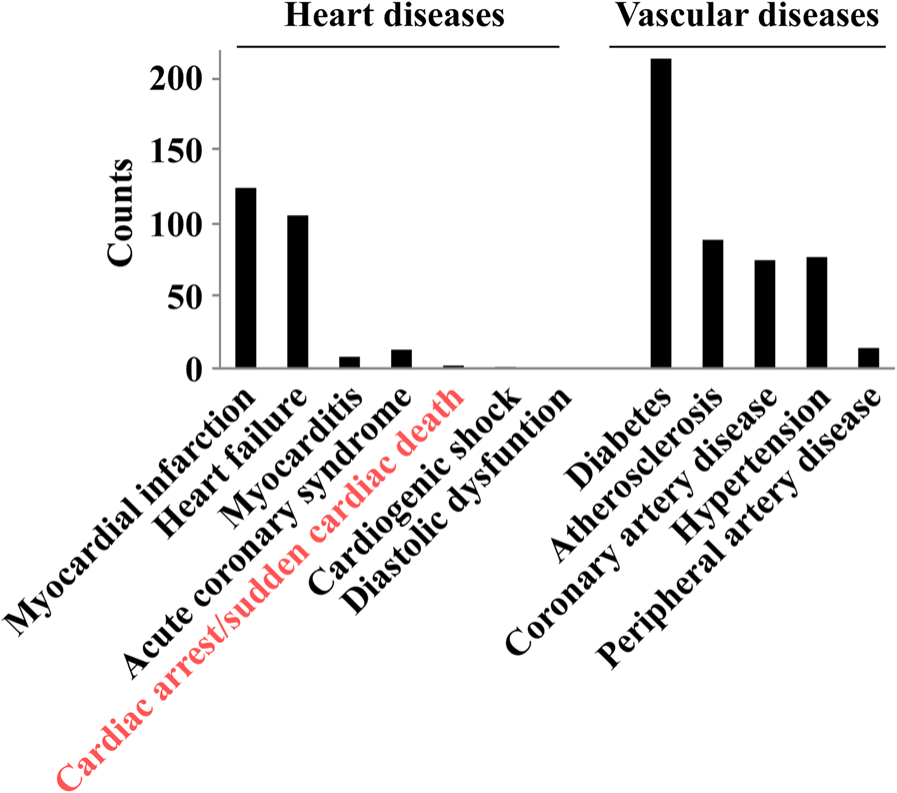
**Numbers of articles related to microRNAs and their biomarker value, according to cardiovascular disease type.** Literature search was performed in PubMed using the query: (microrna OR mirna OR “micro-rna”) AND (biomarker OR diagnostic OR prognostic) AND (name of the specific disease).

A PubMed literature search revealed a plethora of research articles related to miRNAs during the past decade. More than 2,600 articles have been published to date in the field of brain and neurological research (Figure 2A). In the cardiovascular system, 2,300 articles have been published on miRNAs since the first identification of cardiac-enriched miRNAs by Lagos-Quintana and colleagues in 2002 [42] (Figure 2B). Significantly for biomarker research, circulating miRNAs are stable (that is, protected from RNase degradation), and can be easily and accurately quantified using conventional PCR techniques. A PubMed search (query (microrna OR mirna OR “micro-rna”) AND (brain OR neuron OR neurological OR cerebral) AND (biomarker OR diagnostic OR prognostic) showed that, to date, 624 articles were related to miRNAs as potential biomarkers of neurological diseases, the vast majority of which address the value of miRNAs as diagnostic biomarkers of brain tumors. With respect to miRNAs as biomarkers of cardiovascular diseases, we found 724 articles (query (microrna OR mirna OR “micro-rna”) AND (heart OR cardiac OR myocardial OR cardiovascular) AND (biomarker OR diagnostic OR prognostic)), the larger part focusing on myocardial infarction (125 articles) and heart failure (106 articles). Interestingly, only two reports focused on cardiac arrest (Figure 3). Furthermore, the large part of these studies addressed the diagnostic value of miRNAs, not their prognostic value.

Consequently, miRNAs have emerged as candidate biomarkers of brain and heart diseases.

## MicroRNAs and cardiac diseases

Several studies suggested that miRNAs may be used as diagnostic biomarkers of cardiovascular diseases, notably acute myocardial infarction (for recent reviews, see [43-45]). Other reports have addressed the prognostic value of circulating miRNAs after acute myocardial infarction [46-50]. We observed a significant inverse correlation between miR-208b and miR-499 and the left ventricular ejection fraction of patients 4 months after acute myocardial infarction [51]. However, these two miRNAs failed to accurately predict outcome in these patients. In another study involving two independent cohorts of patients with acute myocardial infarction, we found that plasma levels of miR-150, a non-prototypical cardiac miRNA, measured in the first few days following infarction significantly predicts left ventricular remodeling at 4 months [52]. In addition, a combination of several miRNAs, including miR-150, improved the predictive value of brain natriuretic peptide after acute myocardial infarction [53]. While several studies characterized the prognostic value of miRNAs in other cardiovascular conditions such as heart failure, this has not been rigorously investigated in cardiac arrest patients.

## MicroRNAs and brain injury

### MicroRNAs in the ischemic brain

The role of miRNAs in regulating brain development, plasticity and nervous system diseases, including cancer, has been reviewed previously [40,41,54,55]. After cardiac arrest, the brain, as well as other peripheral organs, is subjected to oxygen and nutrient deprivation as a consequence of cessation of blood flow. Expression of miRNAs in the brain is altered following cerebral ischemia [56], and several candidate miRNAs have been identified. In rodent models, miR-233 is up-regulated in ischemic brain and controls the response to neuronal injury by down-regulating the expression of glutamate receptors. This protects neurons from calcium influx mediated by extracellular glutamate accumulation that characterizes the excitotoxicity phase of brain ischemia. A lack of miR-223 leads to memory deficits and neuronal cell death after stroke [57]. The miR-200 and miR-182 families are down-regulated in the brain of hibernating squirrels and inhibition of their activities protects neuronal cells from oxygen and glucose deprivation-induced death [58]. In a rat model of global cerebral ischemia, miR-181c regulates microglial-mediated neuronal apoptosis following ischemia/reperfusion injury [59]. miR-181c directly targets the 3’ untranslated region of TNF-α mRNA, inhibiting apoptosis mediated by TNF-α from activated microglial cells [59]. These data suggest that miRNAs are functionally important mediators of neurological impairment in ischemic brain. Thus, miRNAs may represent not only new biomarkers for neurological prognosis but also novel candidates for neuroprotective therapy targets that may be investigated following cardiac arrest.

### Neuroprotection and microRNAs

A few miRNAs have been identified in therapeutic strategies aiming at protecting the ischemic brain. Valproic acid, a histone deacetylase inhibitor that reduces neurological sequelae and improves motor activity following stroke in rodents [60], regulates miR-331 expression in ischemic neuronal cells [56]. Combined therapy with bortezomib, a proteasome inhibitor approved for treatment of patients with multiple myeloma [61], and tissue plasminogen activator, which is neuroprotective after stroke in aged rats, is associated with an increase of miR-146a expression in cerebral endothelial cells [56,62]. However, whether these miRNAs are neuroprotective *per se* remains to be demonstrated.

### Mild induced hypothermia and microRNAs

Numerous experimental models and two randomized clinical trials have suggested that mild induced hypothermia improves neurological outcome in patients who remain unconscious following out-of-hospital cardiac arrest. This treatment is now standard care in many intensive care units. However, the use of therapeutic hypothermia has been recently challenged by the results of our TTM trial (Target Temperature Management After Cardiac Arrest), which showed that lowering body temperature to 33°C in unconscious survivors of out-of-hospital cardiac arrest did not confer protection compared with 36°C [35]. The putative mechanisms of neuroprotection have been extensively explored in experimental models but there has been little focus on the expression and function of miRNAs. Recent studies have reported that hypothermia regulates miRNAs expression. Truettner and colleagues [63] showed that miRNAs are dysregulated in the brain of hypothermic rats. Pilotte and colleagues [64] showed that hypothermia regulates miRNA expression through enhanced processing of pre-miRNAs by Dicer. The cold-responsive protein Rbm3, a glycine-rich RNA-binding protein, is implicated in disinhibition of Dicer in this process [65]. In pigs subjected to cardiogenic shock, mild induced hypothermia down-regulated plasma levels of miR-122 [66]. Further research is needed to determine whether miRNAs are key players in the neuroprotective effects of cooling. If this could be demonstrated, miRNAs would represent a novel class of neuroprotective agents that would deserve further testing.

### MicroRNAs as therapeutic target

Several lines of evidence support the concept that miRNAs functionally involved in the response of the brain to ischemic injury and miRNAs participating in the neuroprotective effects of hypothermia may be interesting therapeutic targets, either to protect the brain from neurological damage or to stimulate neurological repair after cardiac arrest. This assumption is supported by recent reports, including that of Selvamani and colleagues [67] showing that antagomirs to Let7f or miR-1 are able to extend the neuroprotection afforded by insulin-like growth factor-1 in a rat model of cerebral ischemia. In addition, locked nucleic acid anti-miR130a reduced infarct volume and promoted recovery after transient focal cerebral ischemia in rats [68].

The finding that exosomes conveying miRNAs are able to cross the blood-brain barrier [69] suggests that simple intravenous injection of artificial exosomes may represent an effective way of delivering miRNAs to the ischemic brain. Therefore, miRNAs are promising therapeutic targets that may be further tested, alone or in adjunction with hypothermia, to improve neurological recovery of patients with cardiac arrest.

### MicroRNAs as prognostic biomarkers after cardiac arrest

The biomarker value of miRNAs after cerebral ischemia has been suggested by the observation that specific miRNAs have been detected in the blood after ischemic stroke in both animals [70] and humans [71]. In addition, some of these miRNAs might be potential biomarkers of ischemic stroke [71-73]. As a first attempt to identify miRNAs with prognostic value after cardiac arrest, we performed a proof-of-concept study in which we compared the plasma miRnome of 14 patients with favorable outcome and 14 patients with poor outcome after cardiac arrest [74]. Using microarrays covering almost 700 miRNAs (miRBase release 12.0), we observed a miRNA biosignature linked to outcome. Among miRNAs differentially expressed between patients with favorable outcome and patients with poor outcome, miR-122 and miR-21 were significant predictors of neurological outcome (areas under the receiver-operating characteristic curve of 0.73 and 0.77, respectively) and mortality (*P* < 0.05) at 6 months. We could verify that miR-122 and miR-21 were reliably expressed by neuronal cells, as also shown elsewhere [75,76], comforting our working hypothesis that miRNAs originating from dying neurons after cardiac arrest can be measured in the bloodstream. Consistent with this hypothesis was the demonstration that exosomes, which carry miRNAs outside cells, are able to cross the blood-brain barrier [69]. In addition, disruption of the blood-brain barrier has been shown after cerebral ischemia, which may facilitate the release of neuron-derived miRNAs into the bloodstream [77]. Sheinerman and colleagues [78] identified brain-enriched miRNAs in the blood of patients with mild cognitive impairment, an early stage of multiple neurodegenerative diseases. Thus, brain-derived miRNAs present in the bloodstream after cardiac arrest may indicate neurological damage. Since the extent of neurological damage is a critical determinant of post-cardiac arrest recovery, it is expected that circulating miRNAs may have an interesting prognostic value in this setting. A recent study from our group showing that brain-enriched miR-124 is associated with neurological outcome after cardiac arrest confirmed this assumption [79]. In future studies, the added value of this novel category of biomarkers over existing tools will have to be determined. Also, the sensitivity and specificity of miRNAs, as well as their usefulness for early prediction, will have to outperform current electrophysiological and neuroimaging tools. Finally, the techniques used to quantify miRNAs, which are still time-consuming, will have to be improved, both in terms of reproducibility, rapidity, cost, and standardization.

### Conclusion and future perspectives

The discovery of miRNAs as regulators of gene expression has generated considerable excitement amongst researchers. A number of studies have been conducted addressing their potential as diagnostic, prognostic or therapeutic targets in cerebral and cardiovascular diseases. The value of miRNAs as biomarkers in patients resuscitated following cardiac arrest has, however, received little attention to date. Pilot studies suggest that miRNAs may be useful predictors of neurological outcome and survival after cardiac arrest and adequately powered studies should be undertaken to validate these preliminary findings. Ideally, these studies will determine the optimal time for blood sampling, evaluate the added value of miRNAs over existing prognostic tools, and consider multimarker strategies. Importantly, while it has been shown that cardiac-enriched miRNAs are released very early after cardiac injury [51], the kinetics of release of brain-derived miRNAs after cardiac arrest will have to be accurately characterized. The following main technical issues regarding the measurement of circulating levels of miRNAs will have to be considered: advantages and drawbacks of assessing miRNAs in whole blood versus plasma, and appropriate normalization procedure. Interestingly, miRNAs may function not only as novel biomarkers but also as potential therapeutic targets following cardiac arrest [80].

## Abbreviations

CK-BB: Creatine phosphokinase brain-brain
miRNA: microRNA
NSE: Neuron-specific enolase
TNF: Tumor necrosis factor
